# Adaptive Locomotion Control of a Hexapod Robot via Bio-Inspired Learning

**DOI:** 10.3389/fnbot.2021.627157

**Published:** 2021-01-26

**Authors:** Wenjuan Ouyang, Haozhen Chi, Jiangnan Pang, Wenyu Liang, Qinyuan Ren

**Affiliations:** ^1^College of Control Science and Engineering, Zhejiang University, Hangzhou, China; ^2^Department of Electrical and Computing Engineering, National University of Singapore, Singapore, Singapore

**Keywords:** hexapod robot, two-layer CPG, reinforcement learning, adaptive control, bio-inspired

## Abstract

In this paper, an adaptive locomotion control approach for a hexapod robot is proposed. Inspired from biological neuro control systems, a 3D two-layer artificial center pattern generator (CPG) network is adopted to generate the locomotion of the robot. The first layer of the CPG is responsible for generating several basic locomotion patterns and the functional configuration of this layer is determined through kinematics analysis. The second layer of the CPG controls the limb behavior of the robot to adapt to environment change in a specific locomotion pattern. To enable the adaptability of the limb behavior controller, a reinforcement learning (RL)-based approach is employed to tune the CPG parameters. Owing to symmetrical structure of the robot, only two parameters need to be learned iteratively. Thus, the proposed approach can be used in practice. Finally, both simulations and experiments are conducted to verify the effectiveness of the proposed control approach.

## 1. Introduction

In the past decades, a big step has been taken toward the study of legged robots, such as the study of biped robot (Kim et al., 2020), quadruped robot (Hyun et al., 2014), hexapod robot (Yu et al., 2016), octopod robot (Grzelczyk et al., 2018), and etc. Most of these legged robots have exhibited astonish maneuverabilities in a typically structured environment. Among these legged robots, the hexapod robots have been increasingly attracting attention from scientists and a lot of hexapod robotic prototypes have been successfully developed (Stelzer et al., 2012; Li et al., 2019; Sartoretti et al., 2019; Lele et al., 2020; Zhao and Revzen, 2020). Even though these hexapod robots in shape look very much like the arthropod that the scientists are animating, such as ants or spiders, the robots developed hitherto are still pretty away from real arthropods. One of the main challenges lies in the difficulty of controlling the multi-legs of the robots with coordination to a complex dynamic environment.

To control the locomotion of hexapod robots, from a perspective of cybernetics, two methods are generally adopted, namely kinematics-based and bio-inspired. The former models the locomotion patterns via kinematics analysis. As pointed from the study of Ramdya et al. (2017), three basic locomotion patterns of Drosophila melanogaster have been extracted through biological study, namely tripod locomotion, quadruped locomotion, and five legs support locomotion. Based on the analysis of these three basic locomotion patterns, a foot-force distribution model is established for a hexapod robot walking on an unstructured terrain (Zhang et al., 2014). The study of Zarrouk and Fearing (2015) investigates the kinematics of a hexapod robot using only one actuator and explores the turning issue of the robot. In the work of Sun et al. (2018), the inverse kinematics of an 18-degree-of-freedom (DoF) hexapod robot is calculated to control the dynamicly alternating tripod locomotion of the robot. Since it is hard to accurately model the kinematics of all the six-leg crawling modes, most obtained locomotion patterns from kinematics analysis are rough and trail-and-error strategy is usually necessary for tuning the rough patterns applied on the robots. The study from Delcomyn (1980) indicates that center pattern generators (CPGs), which are mainly located in the central nervous system of vertebrates or in relevant ganglia of invertebrates, are primarily responsible for generating coordinated, rhythmic locomotion patterns of animals in real time, such as crawling, flying, swimming, and running. Inspired by the characteristics of the stability and self-adaption of biological CPGs, artificial CPGs have been extensively studied, namely the bio-inspired approach, for locomotion generation of hexapod robots. The notable examples include the studies in Chung (2015), Zhong et al. (2018), Yu et al. (2020), and Bal (2021). Through these previous studies, it can be found out that the bio-inspired method can greatly simplify the locomotion control problem underlying coordination of multiple legs.

Although the bio-inspired method has been widely and fruitfully applied in locomotion control of many biomimetic robots, it still remains a challenge for modulating the CPG parameters to generate adaptive locomotion for hexapod robots. The CPG parameters in many studies are determined by experiences and some researchers adopt data-driven optimization methods, such as particle swarm optimization (PSO) method and reinforcement learning (RL), to tune the parameters. In the work of Juang et al. (2011), a symbiotic species-based PSO algorithm is proposed to automate the parameter design for evolving dynamic locomotion of a hexapod robot, but reducing the computing complexity of the PSO algorithm is still under research. In addition, the study of Kecskés et al. (2013) points out that PSO method easily suffers from the partial optimism and causes the loss of accuracy in a coordinate system. In locomotion control, there has been recent success in using RL to learn to walk for hexapod robots. In the work of Barfoot (2006), a cooperative Q-learning RL approach is utilized to experimentally learn locomotion for a 6-DoF hexapod robot, but this RL approach may be unable to deal with the hexapod robots that have higher DoF. The researchers in Sartoretti et al. (2019) employ A3C RL algorithm to learn hexapodal locomotion stochastically. Nevertheless, the performance of the learned controller proposed in the study is dependent on a large number of iterations. For the different terrains, the locomotion of a hexapod robot is controlled through training several artificial neural networks via RL method separately (Azayev and Zimmerman, 2020), but the training scenario is limited to the expert policies and thus the adaptivity of the controller may be inflexible for a dynamic environment.

In this paper, a bio-inspired learning approach is proposed for locomotion control of a hexapod robot with environment change. The proposed bio-inspired learning approach can be characterized by the structure of the learning mechanism. Biologists have proved the motor patterns of animals are controlled by neuro systems hierarchically (Fortuna et al., 2004) and functional configuration of CPGs can be regulated according to sensory feedback to produce different motor outputs (Hooper, 2000). Therefore, inspired from biological control systems, a two-layer CPG motion control system is firstly proposed in this paper to generate locomotion for the robot and then the parameters of the CPG tuning issue is explored to enhance the adaptability of the robot. In the proposed bioinspired control system, the outputs of the first layer of the CPG are responsible for generating the basic locomotion patterns, such as tripod locomotion, quadruped locomotion, and five legs support locomotion. The second layer of the CPG acting as a Behavior Layer controls the limb motion of the hexapod robot. In order to adapt to environment change, through sensory feedback, basic locomotion patterns can be switched accordingly, and the limb behavior of the robot is regulated via a RL-based learning approach. Compared to the pure data-driven locomotion control approach, only few of the CPG parameters involved with the limb behavior control need to be learned iteratively. Hence, the proposed locomotion control approach can be adopted to the robot practically.

The rest of this paper is organized as follows. Section 2 introduces the model of the hexapod robot. Section 3 details the two-layer CPG controller and explores its dynamics with numerical studies. Following that, the RL-based learning approach for refining the CPG parameters is presented in section 4. In section 5, both simulations and experiments are conducted to verify the proposed locomotion control approach. Finally, the conclusions and future work are given.

## 2. Modeling of a Hexapod Robot

### 2.1. The Prototype of the Hexapod Robot

**Figure 1:**
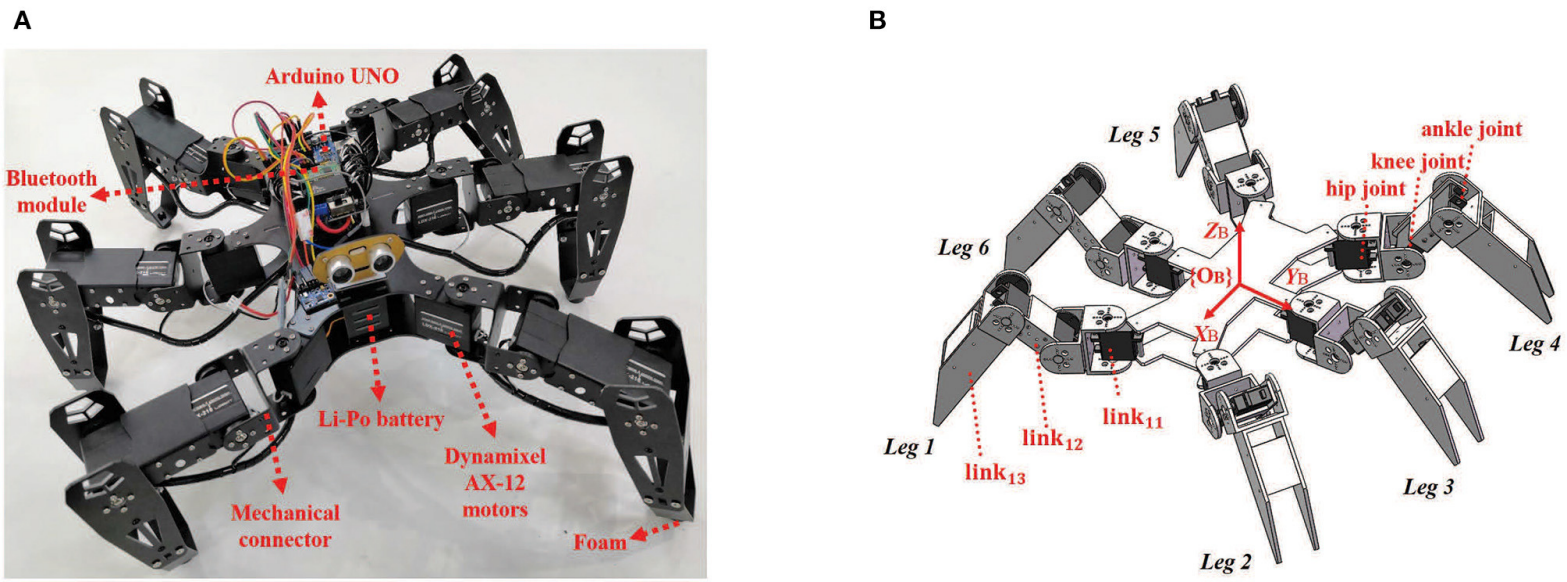
**(A)** The hexapod robot prototype. **(B)** The mechanical schematic of the hexapod robot. {*O*_*B*_} is the body frame whose origin is fixed on the body's mass center. The robot possesses a rectangular body trunk and six mechanical legs numbered from leg 1 to leg 6. There are three joints and three links in each leg. The joints are named as hip joint, knee joint and ankle joint from the direction of body to foot tip. The legs can be labeled as *link*_*i*1_, *link*_*i*2_, *link*_*i*3_ with the leg number *i* = 1, 2, ⋯ , 6. For example, the *link*_11_ (between hip joint and knee joint), *link*_12_ (between knee joint and ankle joint) and *link*_13_ (between ankle joint and foot tip) are the three links of leg 1.

The prototype of the hexapod robot is investigated in this paper shown in Figure 1A, and the specifications are given in Table 1.

**Table 1:** Technical specifications of the prototype.

**Parameter**		**Prototype**	
		**Value**	**Unit**
Number of servo motor		18	\
Power supply		7.4	DC(V)
Total weight		1.995	kg
	Length	24	cm
	Width	18.5	cm
Body dimension	Height	4.5	cm
Limb *link*_*i*1_	Weight	18.6	g
	Length	4.5	cm
	Weight	128	g
Limb *link*_*i*2_	Length	7.5	cm
Limb *link*_*i*3_	Weight	56.3	g
	Length	13.5	cm

*Where i = 1, 2, ⋯ , 6 is the number of six legs*.

Figure 1B illustrates the mechanical schematic of the hexapod robot, which consists of 18 servo motors, a microprocessor, a Bluetooth communication module, a set of mechanical connectors and several other peripherals. Three motors (Dynamixel AX-12) equipped in a leg are concatenated together to act as three joints. A microprocessor (Arduino UNO) is used for processing sensor data, transferring diagnostic information via the Bluetooth module, making decisions and controlling servo motors. Besides that, an external camera (Logitech C930) is employed to track the position of the robot as feedback signals.

### 2.2. Modeling

**Figure 2:**
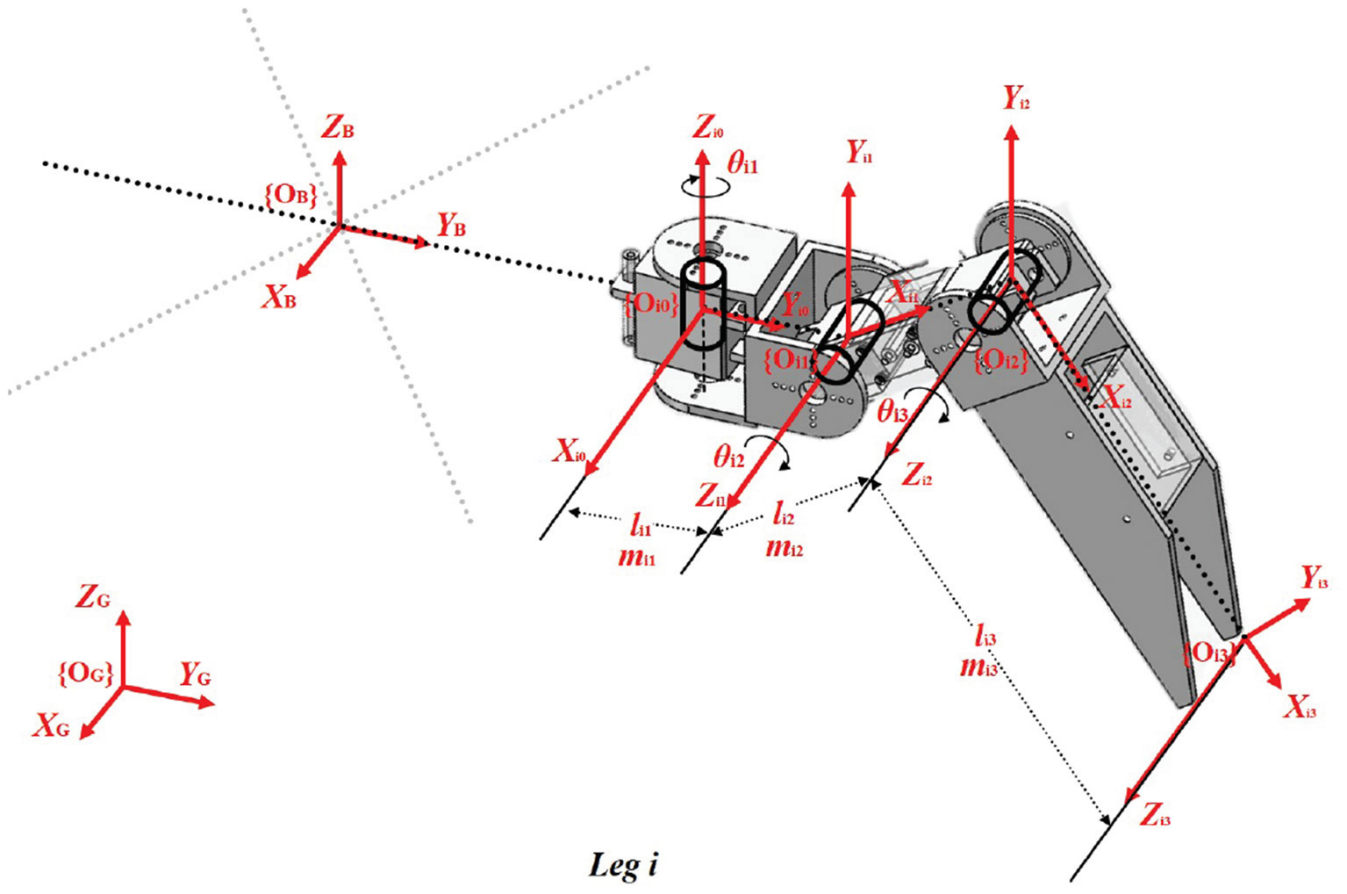
Coordinates at different joints of each leg *i*. {*O*_*G*_} denotes the global coordinate, and {*O*_*ik*_} (*k* = 0, 1, 2, 3) represents the floating frame whose origin fixed on the joints or the foot tip. *l*_*i*1_, *l*_*i*2_, *l*_*i*3_ and *m*_*i*1_, *m*_*i*2_, *m*_*i*3_ are the length and mass of the leg links, respectively. And θ_*i*1_, θ_*i*2_, θ_*i*3_ are the rotational joint angles around *Z*_*i*0_, *Z*_*i*1_, *Z*_*i*2_ axis of the leg.

To establish the kinematic/dynamic model of the hexapod robot, the joint coordinates of each leg *i* are defined as depicted in Figure 2.

The kinematic model is represented by Denavit-Hartenberg (DH) parameters for resolving inverse kinematic of the leg. According to these fixed frames, the transformation parameters and DH parameters are demonstrated in Tables 2, 3, respectively.

**Table 2:** Transformation parameters from the {*O*_*i*0_} to the {*O*_*B*_}.

**Leg *i***	**1**	**2**	**3**	**4**	**5**	**6**
*d*_*xi*_(*mm*)	33.5	67	33.5	−33.5	−67	−33.5
*d*_*yi*_(*mm*)	58	0	−58	−58	0	58
$\varphi_{i}{(}^{{}^{\circ}})$	−60	0	60	120	180	−120

*The hip joint position is defined as (d_xi_, d_yi_) and φ_i_ denotes the direction angle in the body frame {O_B_}*.

**Table 3:** Denavit-Hartenberg parameters.

**Joint j**	**α_*ij*_**	**a_*ij*_**	**d_*ij*_**	**θ_*ij*_**
1	π/2	*l*_*i*1_	0	θ_*i*1_
2	0	*l*_*i*2_	0	θ_*i*2_
3	0	*l*_*i*3_	0	θ_*i*3_

*Where j = 1,2,3 is the joint number from hip joint to knee joint of each leg. And α_ij_ is the link twist indicating the angle from Z_i(k−1)_ to Z_ik_ around X_ik_, a_ij_ is the link length representing the distance from the intersection of Z_i(k−1)_ and X_ik_ to the origin of X_ik_, d_ij_ is the joint distance meaning the distance from the intersection of Z_i(k−1)_ and X_ik_ to the origin of Z_i(k−1)_, θ_ij_ is the joint angle showing the angle from X_i(k−1)_ to X_ik_ around Z_i(k−1)_*.

The relative translation and rotation between the (*j* − 1)*th* and the *jth* joint coordinates are computed by the transformation matrix (1):

(1) $${}^{i}T_{j}^{j-1}=\left[\begin{array}{cccc} \cos\theta_{ij} & -\cos\alpha_{ij}\sin\theta_{ij} & \sin\alpha_{ij}\sin\theta_{ij} & a_{ij}\cos\theta_{ij}\\ \sin\theta_{ij} & \cos\alpha_{ij}\cos\theta_{ij} & -\sin\alpha_{ij}\cos\theta_{ij} & a_{ij}\sin\theta_{ij}\\ 0 & \sin\alpha_{ij} & \cos\alpha_{ij} & d_{ij}\\ 0 & 0 & 0 & 1 \end{array}\right],$$

where especially, the transition matrix between the body coordinate {*O*_*B*_} and the hip joint coordinate {*O*_*i*0_} is represented by (2):

(2) $${}^{i}T_{0}^{B}=\left[\begin{array}{cccc} \cos\varphi_{i} & -\sin\varphi_{i} & 0 & d_{xi}\\ \sin\varphi_{i} & \cos\varphi_{i} & 0 & d_{yi}\\ 0 & 0 & 1 & 0\\ 0 & 0 & 0 & 1 \end{array}\right].$$

Consequently, the foot tip coordinate {*O*_*i*3_} can be transformed into the body coordinate {*O*_*B*_} by multiplying the previous matrixs sequentially shown in (3):

(3) $$\begin{array}{l} {}^{i}T_{3}^{B}={\;}^{i}T_{0}^{B}\cdot^{i}T_{1}^{0}\cdot^{i}T_{2}^{1}\cdot^{i}T_{3}^{2}\\ \;=\;\left[\begin{array}{cccc} \cos(\varphi_{i}+\theta_{i1})\;\cos(\theta_{i2}+\theta_{i3}) & -\cos(\varphi_{i}+\theta_{i1})\;\sin(\theta_{i2}+\theta_{i3}) & \sin(\varphi_{i}+\theta_{i1}) & d_{xi}+\cos(\varphi_{i}+\theta_{i1})(l_{i1}+l_{i2}\cos(\theta_{i2})+l_{i3}\cos(\theta_{i2}+\theta_{i3}))\\ \sin(\varphi_{i}+\theta_{i1})\;\cos(\theta_{i2}+\theta_{i3}) & -\sin(\varphi_{i}+\theta_{i1})\;\sin(\theta_{i2}+\theta_{i3}) & -\cos(\varphi_{i}+\theta_{i1}) & d_{yi}+\sin(\varphi_{i}+\theta_{i1})(l_{i1}+l_{i2}\cos(\theta_{i2})+l_{i3}\cos(\theta_{i2}+\theta_{i3})\\ \sin(\theta_{i2}+\theta_{i3}) & \cos(\theta_{i2}+\theta_{i3}) & 0 & l_{i2}\sin(\theta_{i2})+l_{i3}\sin(\theta_{i2}+\theta_{i3})\\ 0 & 0 & 0 & 1 \end{array}\right].\; \end{array}$$

Thus, the position of the foot tip with respect to the body coordinate {*O*_*B*_} can be derived as given below:

(4) $$\begin{array}{l} \left[\begin{array}{c} p_{xi}\\ p_{yi}\\ p_{zi} \end{array}\right]\\ =\;\left[\begin{array}{c} d_{xi}+\cos(\varphi_{i}+\theta_{i1})(l_{i1}+l_{i2}\cos(\theta_{i2})+l_{i3}\cos(\theta_{i2}+\theta_{i3})\\ d_{yi}+\sin(\varphi_{i}+\theta_{i1})(l_{i1}+l_{i2}\cos(\theta_{i2})+l_{i3}\cos(\theta_{i2}+\theta_{i3})\\ l_{i2}\sin(\theta_{i2})+l_{i3}\sin(\theta_{i2}+\theta_{i3}) \end{array}\right], \end{array}$$

where ${\left[p_{xi}\;p_{yi}\;p_{zi}\right]}^{T}$ is the position coordinate of the *ith* foot hip and θ_*ij*_ is the joint angle.

The leg of the hexapod robot is a complex joint-link system connecting the body trunk with the ground. Hence, closed kinematics chains can be found in the robot system. Since forces and moments propagate via the kinematics chains among different legs (Roy and Pratihar, 2013), the kinematics and dynamics are coupled. The dynamic model of such a coupled hexapod robot with 18 actuators is derived via Lagrangian-Euler method as follows:

(5) $$\begin{array}{r} \tau_{i}={\text{M}}_{i}\left(\theta \right){\ddot{\theta }}_{i}+{\text{H}}_{i}\left(\theta ,\dot{\theta }\right){\dot{\theta }}_{i}+{\text{G}}_{i}\left(\theta \right)-{\bar{\text{J}}}_{i}^{T}{\text{F}}_{i}\;, \end{array}$$

where $\tau_{i}={\left[\tau_{i1}\;\tau_{i2}\;\tau_{i3}\right]}^{T}\in {\mathbb{R}}^{3}$ is the joint torque vector of the *ith* leg consisting of hip joint torque τ_*i*1_, knee joint torque τ_*i*2_ and ankle joint torque τ_*i*3_. $\theta_{i}={\left[\theta_{i1}\;\theta_{i2}\;\theta_{i3}\right]}^{T}\in {\mathbb{R}}^{3},{\dot{\theta }}_{i}={\left[{\dot{\theta }}_{i1}\;{\dot{\theta }}_{i2}\;{\dot{\theta }}_{i3}\right]}^{T}\in {\mathbb{R}}^{3},{\ddot{\theta }}_{i}={\left[{\ddot{\theta }}_{i1}\;{\ddot{\theta }}_{i2}\;{\ddot{\theta }}_{i3}\right]}^{T}\in {\mathbb{R}}^{3}$ are joint angle, joint angle acceleration, and joint angle jerk vector of the *ith* leg, respectively. ${\text{M}}_{i}\left(\theta \right)\in {\mathbb{R}}^{3\times 3}$ is a inertia matrix of the *ith* leg. ${\text{H}}_{i}\left(\theta ,\dot{\theta }\right)\in {\mathbb{R}}^{3\times 3}$ is Coriolis forces matrix of the *ith* leg. ${\text{G}}_{i}\left(\theta \right)\in {\mathbb{R}}^{3}$ is a link gravitational forces vector of the *ith* leg. ${\text{F}}_{i}={\left[f_{ix}\;f_{iy}\;f_{iz}\right]}^{T}\in {\mathbb{R}}^{3}$ represents ground reaction forces of the *ith* support foot tip with the coordinate {*O*_*i*3_}. ${\bar{\text{J}}}_{i}\in {\mathbb{R}}^{3\times 3}$ is the Jacobian matrix of the *ith* leg, computed by (6). Moreover, the position and velocity of the hexapod robot in this work are transformed to the global coordinate {*O*_*G*_}.

(6) $${\bar{J}}_{i}=\left[\begin{array}{ccc} -(l_{i1}+l_{i2}\cos(\theta_{i2})+l_{i3}\cos(\theta_{i2}+\theta_{i3})\;\sin(\theta_{i1}) & -(l_{i2}\sin(\theta_{i2})+l_{i3}\sin(\theta_{i2}+\theta_{i3}))\;\cos(\theta_{i1}) & -l_{i3}\sin(\theta_{i2}+\theta_{i3})\;\cos(\theta_{i1})\\ \left(l_{i1}+l_{i2}\cos(\theta_{i2})+l_{i3}\cos(\theta_{i2}+\theta_{i3})\;\cos(\theta_{i1}\right) & -(l_{i2}\sin(\theta_{i2})+l_{i3}\sin(\theta_{i2}+\theta_{i3}))\;\sin(\theta_{i1}) & -l_{i3}\sin(\theta_{i2}+\theta_{i3})\;\sin(\theta_{i1})\\ 0 & l_{i2}\cos(\theta_{i2})+l_{i3}\cos(\theta_{i2}+\theta_{i3}) & l_{i3}\cos(\theta_{i2}+\theta_{i3}) \end{array}\right].$$

## 3. Locomotion Controller via CPG

**Figure 3:**
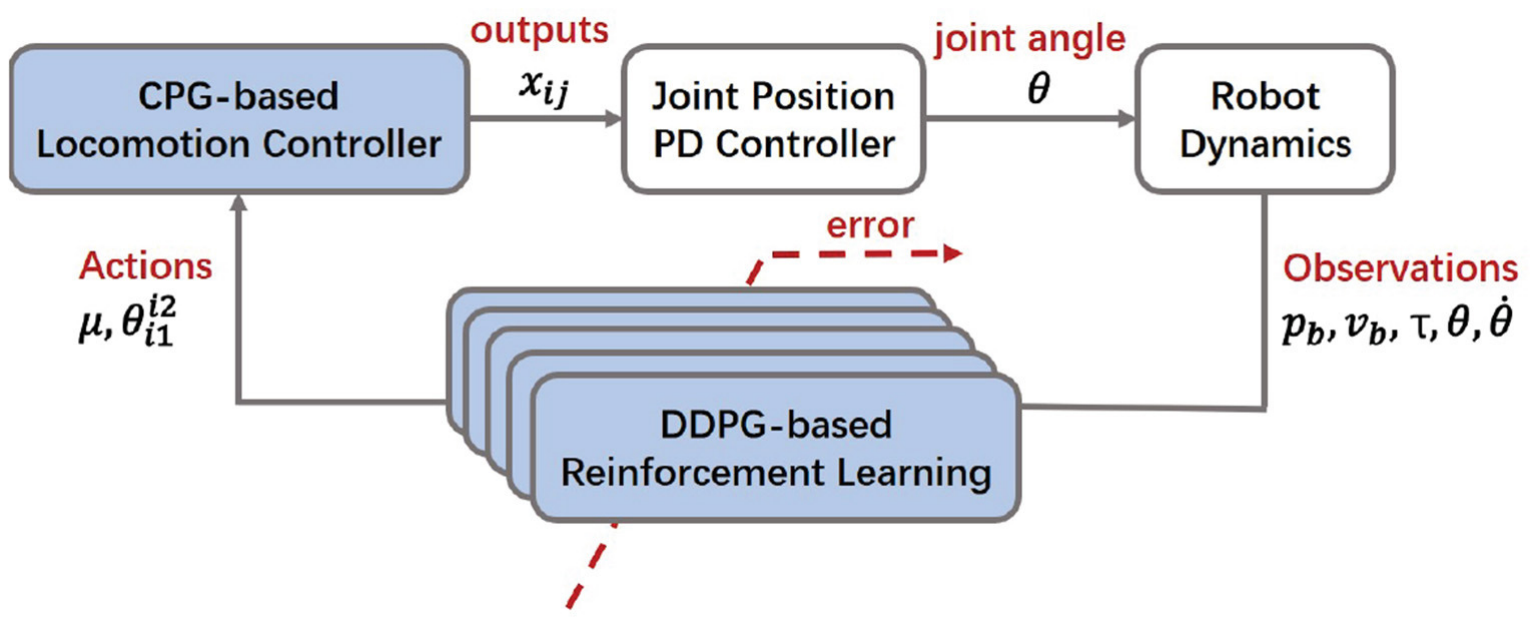
Diagram of the proposed bio-inspired control scheme. The proposed control scheme has a cascaded structure with a feedback loop. It consists of three parts: (1) A dynamic model (with a embedded PD controller) that computes torque commands to handle robot dynamics subject to mechanical constraints. The dynamics parameters $p_{b}={\left[p_{x},p_{y},p_{z}\right]}^{T}$, $v_{b}={\left[v_{x},v_{y},v_{z}\right]}^{T}$ are the robot body position and velocity vector,respectively; τ, θ, $\dot{\theta }$ indicate the joint torque, angle and angle velocity, respectively. (2) A two-layer CPG locomotion controller that outputs coordinated signals to generate the basic locomotion. The CPG parameters μ and $\theta_{i1}^{i2}$ are the inputs representing the amplitude and the phase difference between the hip joint *i*1 and the knee joint *i*2 of the leg i, respectively; *x*_*ij*_ is the output signal. (3) A DDPG-based RL motion controller that trains the optimal locomotion via the cost function.

Based on the analysis of the aforementioned mathematical model, the whole control scheme is proposed as shown in Figure 3. Inspired by biological arthropods, a hexapod robot is supposed to exhibit various locomotion in different terrains, such as tripod locomotion, quadruped locomotion, and five legs support locomotion (Zhong et al., 2018). Among these locomotion patterns, the tripod locomotion can achieve the fastest movement, while the quadruped and five legs support locomotion are more flexible. In this work, the locomotion patterns can be judged by velocity criterion according to the change of terrains.

**Figure 4:**
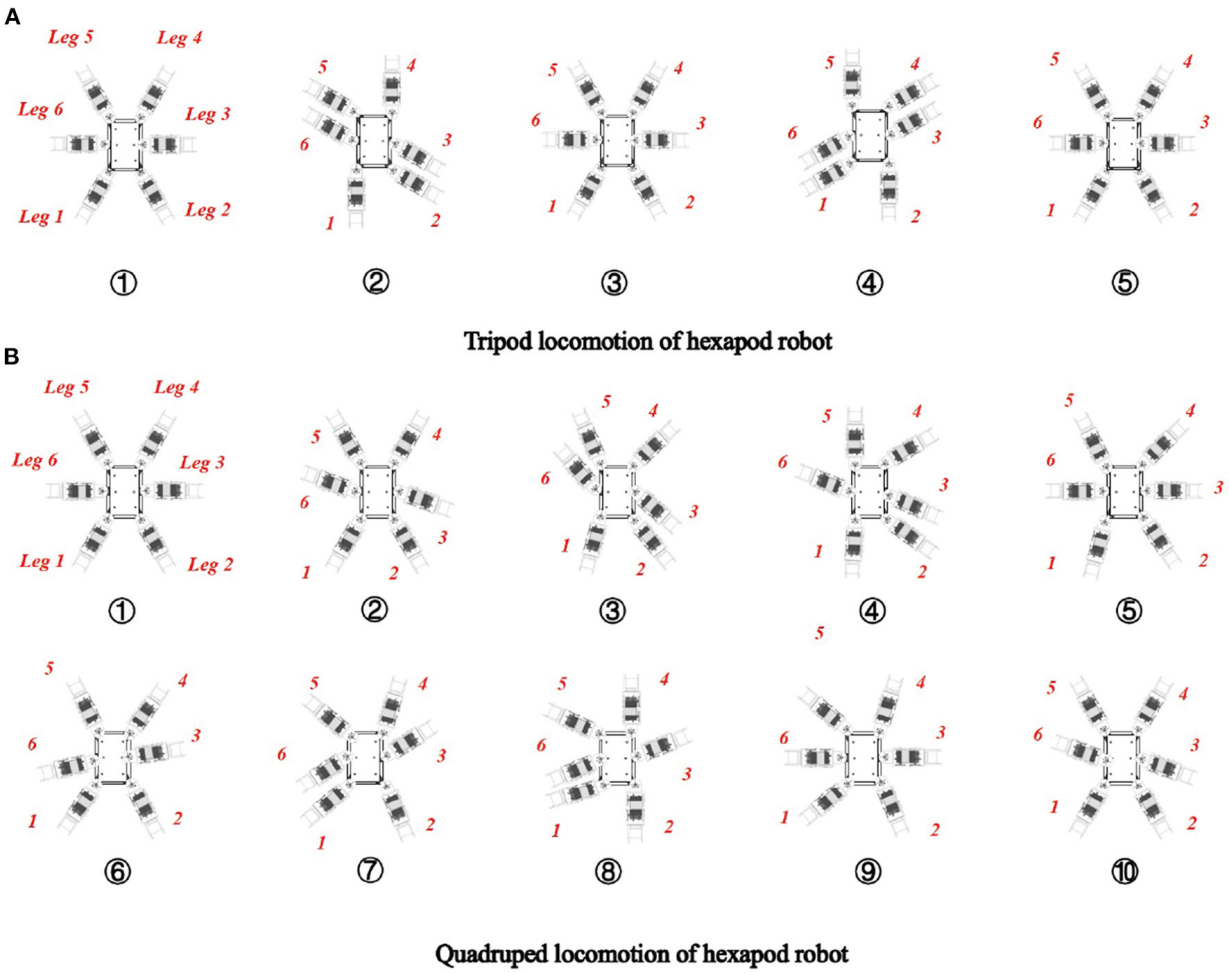
Locomotion patterns via the body layer. During each cycle, six legs in the tripod locomotion are separated into two sets of {*leg* 1, *leg* 3, *leg* 5} and {*leg* 2, *leg* 4, *leg* 6} moving alternately, while in the quadruped locomotion are separated into four sets of {*leg* 3}, {*leg* 1, *leg* 4}, {*leg* 6}, and {*leg* 2, *leg* 5} to move successively.

In nature, CPGs are mainly used for generating coordinated and rhythmic movements for the locomotion of animals. Based on the similarity between biological legged animals and hexapod robots as well as the attractive capability of the CPG-based model on coupling the dynamics of robots, artificial CPG-based locomotion controllers are widely adopted to generate the locomotion behaviors of the biological counterparts. The basic locomotion patterns of the hexapod robot and the phase relations of the locomotion patterns are illustrated in Figures 4A,B.

### 3.1. Two-Layer CPGs Model

Due to complicated couplings and high degrees of freedom on the hexapod robot, the proposed CPG-based locomotion control is decomposed into two layers: (1) The body layer consists of six hip oscillators with bidirectional couplings. (2) The limb layer includes three oscillators in association with the hip joint, the knee joint and the ankle joint in every leg, where the knee joint oscillator and ankle joint oscillator are interconnected with bidirectional coupling, but the oscillator pair is unidirectionally controlled by the corresponding hip oscillator in the body layer.

**Figure 5:**
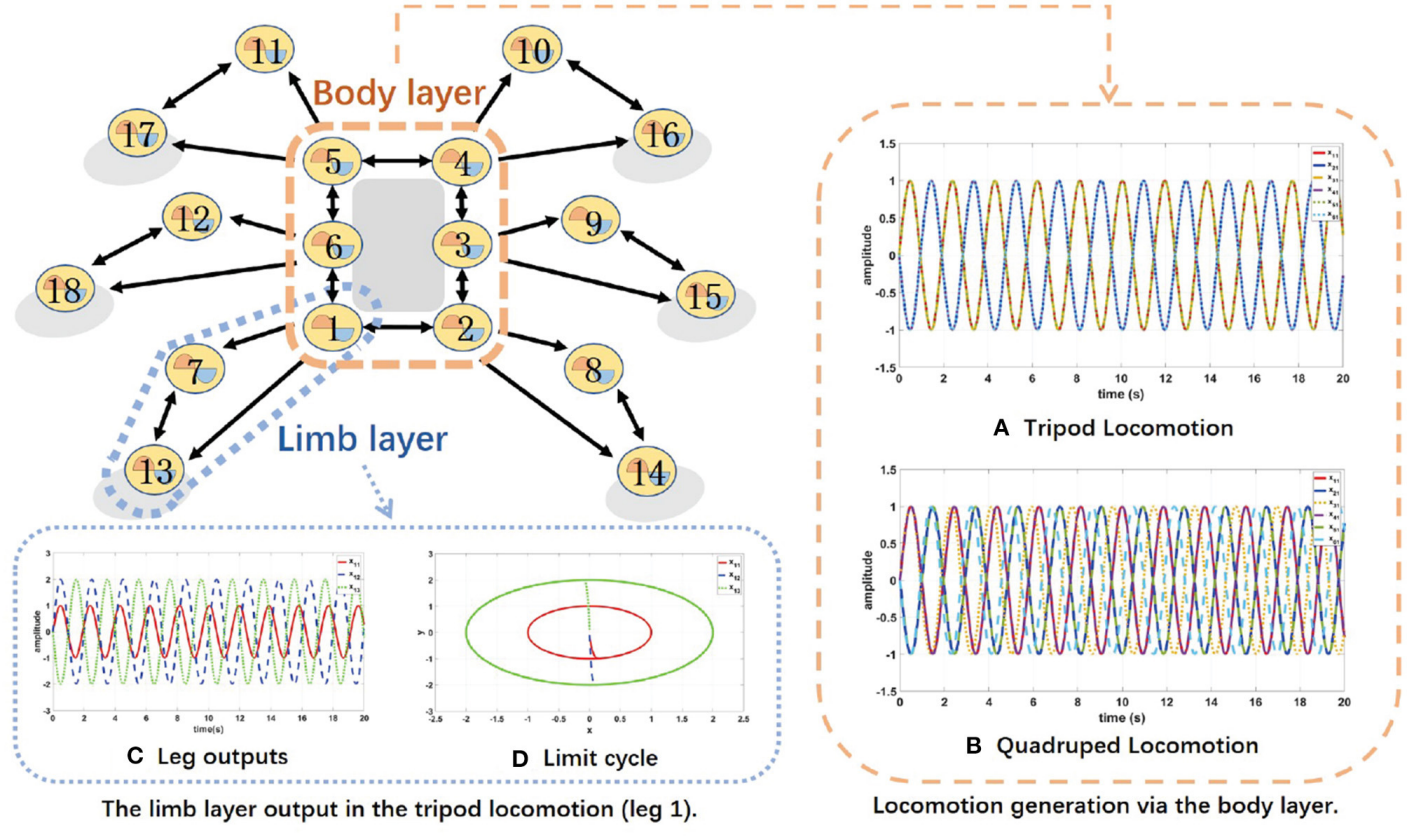
The topology network of the proposed two-layer CPG.

Therefore, the body layer acting as a Conscious Layer shown in Figures 5A,B provides knowledge to determine the locomotion mode of the hexapod robot, while the limb layer acting as a Behavior Layer shown in Figures 5C,D has a major impact on final motion states and performance.

Considering the stable limit cycle and the explicit interpretable parameters, Hopf oscillator is a suitable element to construct CPGs for robotic locomotion (Seo et al., 2010). Hence, in this work, our CPG model can be described as a set of coupled Hopf oscillators and each Hopf oscillator is formulated by (7):

(7) $$\left\{ \begin{array}{l} \dot{x}=\alpha (\mu^{2}-x^{2}-y^{2})x-\omega y\\ \dot{y}=\beta (\mu^{2}-x^{2}-y^{2})y-\omega x \end{array}\right.,$$

where *x* and *y* are two state variables, ω is the frequency, α and β are the positive constants which determine the convergence rate of the limit cycle. In this paper, *x* is defined as the output signal of the oscillator.

Since the hexapod robot in this work has six legs and each leg has 3 DoF, a network consisted of 18 Hopf oscillators is proposed. According to the proposed CPG model shown in Figure 5, to achieve desired motion of the hexapod robot, multiple oscillators are needed to be coupled together to guarantee robotic system synchronization and coordination. Motivated by the work presented by Campos et al. (2010), the proposed CPG model connected by the diffusive coupling is described by:

(8) $$\begin{array}{l} \left[\begin{array}{c} {\dot{x}}_{ij}\\ {\dot{y}}_{ij} \end{array}\right]\;=\left[\begin{array}{cc} \alpha (\mu^{2}-x_{ij}^{2}-y_{ij}^{2}) & -\omega_{ij}\\ \omega_{ij} & \beta (\mu^{2}-x_{ij}^{2}-y_{ij}^{2})y_{ij} \end{array}\right]\left[\begin{array}{c} x_{ij}\\ y_{ij} \end{array}\right]\\ \;+\;k\cdot \;\sum_{mn\neq ij}\bar{R}(\theta_{mn}^{ij})\left[\begin{array}{c} 0\\ \frac{x_{mn}+y_{mn}}{\sqrt{x_{mn}^{2}+y_{mn}^{2}}} \end{array}\right], \end{array}$$

where *x*_*ij*_, *y*_*ij*_ with *i* = 1, 2, ⋯ , 6 and *j* = 1, 2, 3 denote two state variables. The constant coupling strength *k* = 0.1 and the convergence coefficients α = β = 100 are set for all oscillators, which are determined through a trial-and-error simulation on the stability of limit cycle in this work. The oscillator frequencies are unified as ω_*ij*_ = ω for simplifying the high-level optimization. Besides, $\theta_{mn}^{ij}$ with *m* = 1, 2, ⋯ , 6 and *n* = 1, 2, 3 represents the phase difference between the joint *ij* and the joint *mn*, then an associated 2D rotation matrix $\bar{R}\left(\theta_{mn}^{ij}\right)$ is defined as:

(9) $$\begin{array}{r} \bar{R}\left(\theta_{mn}^{ij}\right)=\left[\begin{array}{cc} \cos\left(\theta_{mn}^{ij}\right) & -\sin\left(\theta_{mn}^{ij}\right)\\ \sin\left(\theta_{mn}^{ij}\right) & \cos\left(\theta_{mn}^{ij}\right) \end{array}\right]. \end{array}$$

Compared with (7), the coupling relations among different Hopf oscillators are embedded into the artificial CPG model. This proposed 3D two-layer CPG model not only can regulate the basic locomotion patterns of the hexapod robot, but also fine-tune the motion performance for adapting to environment change. More information about the superiority of the 3D topology are demonstrated in our previous work (Niu et al., 2014). Through this CPG-based locomotion controller, the coordination can be adjusted with fewer parameters, which effectively reduce the control dimension and complexity of the system.

### 3.2. Simulation of Locomotion Generation

To verify the performance of the proposed CPG-based locomotion controller, several simulations are conducted. In the first layer of the network, the phase differences of the body layer among different hip joints are set as shown in Table 4 to generate the tripod locomotion or quadruped locomotion. The six body oscillator parameters in the tripod and quadruped locomotion are set as *amplitude* = 1 and *frequency* = 3.14.

**Table 4:** Phase differences in corresponding locomotion.

**Phase differences**	**Locomotion patterns**			
	**Tripod** ***(deg)***		**Quadruped** ***(deg)***	
θ_11_	0	$\theta_{mn}^{ij}=360\cdot \left[\begin{array}{cccccc} 0 & \frac{1}{2} & 0 & 0 & 0 & \frac{1}{2}\\ -\frac{1}{2} & 0 & -\frac{1}{2} & 0 & 0 & 0\\ 0 & \frac{1}{2} & 0 & \frac{1}{2} & 0 & 0\\ 0 & 0 & -\frac{1}{2} & 0 & -\frac{1}{2} & 0\\ 0 & 0 & 0 & \frac{1}{2} & 0 & \frac{1}{2}\\ -\frac{1}{2} & 0 & 0 & 0 & -\frac{1}{2} & 0 \end{array}\right]$	0	$\theta_{mn}^{ij}=360\cdot \left[\begin{array}{cccccc} 0 & \frac{2}{4} & \frac{1}{4} & 0 & \frac{2}{4} & \frac{3}{4}\\ -\frac{2}{4} & 0 & -\frac{1}{4} & -\frac{2}{4} & 0 & \frac{1}{4}\\ -\frac{1}{4} & \frac{1}{4} & 0 & -\frac{1}{4} & \frac{1}{4} & \frac{2}{4}\\ 0 & \frac{2}{4} & \frac{1}{4} & 0 & \frac{2}{4} & \frac{3}{4}\\ -\frac{2}{4} & 0 & -\frac{1}{4} & -\frac{2}{4} & 0 & \frac{1}{4}\\ -\frac{3}{4} & -\frac{1}{4} & -\frac{2}{4} & -\frac{3}{4} & -\frac{1}{4} & 0 \end{array}\right]$
θ_21_	180		180	
θ_31_	0		90	
θ_41_	180		0	
θ_51_	0		180	
θ_61_	180		270	

As can be seen from Figures 5A,B, the outputs of the body layer network are stable and periodic, while the phase differences between the neighboring oscillators maintain strictly 180*deg* for tripod locomotion and 90*deg* for quadruped locomotion, respectively.

Take the tripod locomotion patterns in leg 1 as an example, the limb layer network firstly receives the corresponding hip joint signal from the body layer. Secondly, the limb network outputs two signals to control the knee joint and the ankle joint interacting with environment. The phase difference between the knee joint and the ankle joint is limited to 180 *deg* in each leg with *amplitude* = 2 and *frequency* = 3.14.

As shown in Figure 5C, the phase difference between the knee joint and the ankle joint is locked in the limb layer. Moreover, Figure 5D presents the stable limit cycle of the coupled Hopf oscillators, which alleviates the influence of disturbances and ensures the smooth tuning of the robot locomotion. These simulation results show that the proposed CPG-based locomotion controller carry the potential of excellent controllability and robustness in unknown and unstructured terrains via online adjustment.

It should be noted that several parameters play important roles in the two-layer CPG controller, namely, amplitudes, frequencies, and phase differences. The CPG allows direct modulation of these parameters to enhance locomotion adaptability of the hexapod robot, but the manual tuning process still remain a challenge. Motivated by the movement of the six-legged arthropods modulated further via higher controller from brain-stem level (Yu et al., 2020), a RL-based controller is proposed to optimize the specialized locomotion patterns automatically in the next section.

## 4. Locomotion Optimization via Reinforcement Learning

### 4.1. Problem Statement

Locomotion of a hexapod robot can be considered as a Markov Decision Process (MDP), which is described as an agent interacting with the environment in discrete time steps. At each time step *t*, the state of the agent and the environment can be jointly described by a state vector *s* ∈ *S*, where *S* is the state space. The agent takes an action *a*_*t*_ ∈ *A*, after which the environment advances a single time step and reaches a new state *s*_*t*+1_ through an environment state-transition distribution P : *S* × *A* × *S* → [0, 1]. Each state-action transition process is evaluated by a scalar reward function R : *S* × *A* → ℝ. At the beginning of each episode, the agent finds itself in a random initial state *s*_0_ ~ ρ(*s*). Thus, the MDP is defined as a tuple (*S, A*, R, P, ρ) (Tan et al., 2018).

In the MDP, the agent selects the action *a* under the state *s* through a stationary policy π : *S* → *P*(*A*), which is defined as a function mapping states into probability distributions over actions. The set of all stationary policies is denoted as Π. Give a performance measurement function as:

(10) $$J(\pi )=\underset{\varsigma ∼\pi }{E}[\sum_{t=0}^{\infty }\gamma^{t}R(s_{t},a_{t},s_{t+1})],$$

where γ ∈ [0, 1) is the discount factor and ς denotes a trajectory under the policy π. The objective of the RL is to select a optimal policy π^*^ that maximizes *J*(π), i.e.,

(11) $$\pi^{*}=\underset{\pi \in \;\Pi }{argmax}\;J(\pi ).$$

However, lack of complete freedoms when training the hexapod robot could suffer from some failed actions, such as collisions, falls, and inaccessible locomotion for a real robot. To tackle these issues, the actions of the hexapod robot should be constrained by several conditions such as acceleration, velocity, and torque constraints, which ensures the robot safe exploration.

Similar to the MDP, a constrained Markov Decision Process (CMDP) is defined as a tuple (*S, A*, R, *C*, P, *d*, ρ). The difference between the MDP and the CMDP is that the policies are trained under additional cost constrains *C* in the latter. Each cost function *C*_*l*_ : *S* × *A* × *S* → ℝ maps transition tuples into costs with the limit *c*_*l*_. Thus, the discounted cost of policy π with cost function *C*_*l*_ (Achiam et al., 2017) is represented by:

(12) $$J_{C_{l}}(\pi )=\underset{\varsigma ∼\pi }{E}[\sum_{t=0}^{\infty }\gamma^{t}C_{l}(s_{t},a_{t},s_{t+1})].$$

where *l* is the number of the constraints.

The set of feasible stationary policies in a CMDP is

(13) $$\Pi_{C}=\{\pi \in \Pi :\forall l,\;J_{C_{l}}(\pi )<=c_{l}\},$$

and the reinforcement learning problem in a CMDP is formulated as:

(14) $$\pi^{*}=\underset{\pi \in \;\Pi_{C}}{argmax}\;J(\pi ).$$

### 4.2. Deep Deterministic Policy Gradient Algorithm

**Figure 6:**
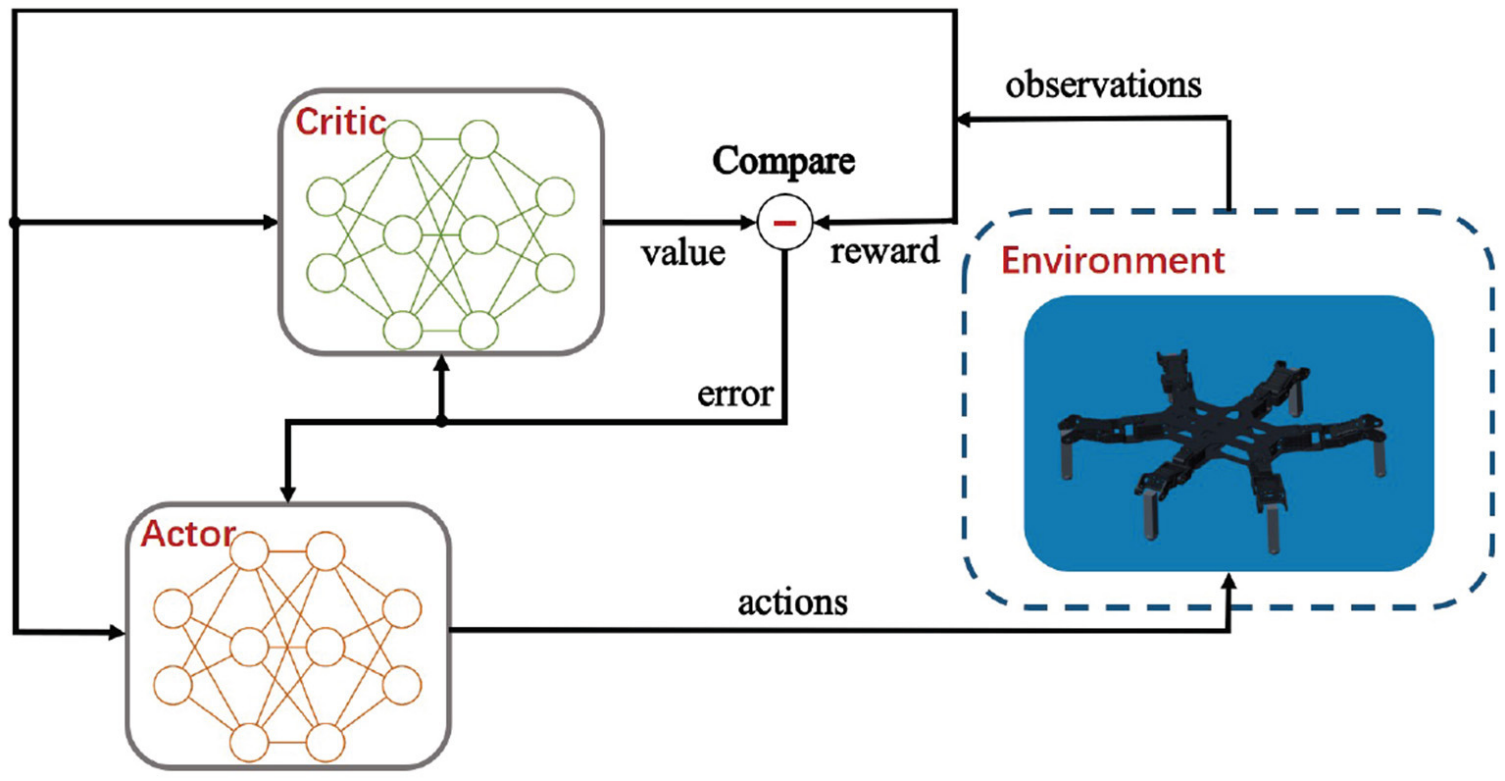
The DDPG-based reinforcement learning structure.

Hexapod robots are multiple-input-multiple-output (MIMO) systems, so generally both the state space and the action space are high-dimensional and continuous. While many of stochastic policy gradient-based RL methods require massive and time-consuming search in such a vast space, deterministic policy greatly improve learning rates without sampling in the action space. Deep deterministic policy gradient (DDPG) (Lillicrap et al., 2016) as a model-free, off-policy RL algorithm, which could deal with unprocessed, high-dimensional sensory inputs and learn policies in a high-dimensional continuous action space via deep function approximators, has been widely accepted for robot control. Adaptive locomotion control of a hexapod robot is a challenging task due to the high-dimensional observations and continuous actions. In this work, the DDPG-based reinforcement learning optimization approach is proposed and illustrated in Figure 6.

Significantly, the DDPG combines an actor-critic method with deep neural networks (DNNs), and it shows stable performance in tough physical control problems including complex multi-joint movements and unstable contact dynamics. Besides, compared with the on-policy and stochastic counterparts such as proximal policy optimization (PPO), the off-policy and deterministic feature of DDPG ensures a more sample-efficient learning owing to the ability of generating a deterministic action.

The proposed DDPG algorithm is applied on learning the adaptive control policy π for the hexapod robot. The control policy π is assumed to be parameterized by θ^π^. Specifically, the RL problem of learning the optimal control policy is converted into learning the optimal value θ^π^. Considering that Policy Gradient is utilized for continuous action space, DDPG algorithm actually combines Policy Gradient with an actor-critic network. The parameter vector θ^π^ is updated in the direction that maximizes the performance measurement function *J*(π). The direction, defined as the action gradient, is the gradient of *J*(π) with respect to θ^π^ which can be calculated as follows:

(15) $$\begin{array}{r} \nabla_{\theta^{\pi }}J\left(\pi \right)=E\left[\nabla_{a}Q\left(s,a\right)\nabla_{\theta^{\pi }}\pi \left(s\right)\right], \end{array}$$

where the action gradient of the performance measurement function *J*(π) depends on the action-value function *Q*(*s, a*), which is unknown and need to be estimated. To achieve the estimation, a critic network *Q* parameterized by θ^*Q*^ is used to approximate the action-value function and an actor network based on the current state offers control policy π that outputs the deterministic action in continuous space. In DDPG, the actor network and critic network are approximated by DNNs which can be learned via policy gradient method and error back propagation, respectively.

The use of neural networks for learning action-value function and control policy tends to be unstable. Thus, DDPG employs two important ideas to solve this problem.

Remark 1. *A copy of the critic network and actor network: a target critic network and a target actor network are constructed and parameterized by vector θ^*Q*^′ and θ^π^′, respectively*. *These two target networks are adopted to calculate the target values, and the parameters Q*′**and π**′ *in the two target networks slowly track the parameters Q and π in the original critic and actor network as follows:*

(16) $$\begin{array}{r} \theta^{Q^{\prime }}=\kappa \theta^{Q}+\left(1-\kappa \right)\theta^{Q^{\prime }}, \end{array}$$

(17) $$\begin{array}{r} \theta^{\pi^{\prime }}=\kappa \theta^{\pi }+\left(1-\kappa \right)\theta^{\pi^{\prime }}, \end{array}$$

*where κ is positive and κ* << 1. *The updating mechanism is called soft update, which avoids non-stationary target values and enhances the stability*.

Remark 2. *Another challenge using neural networks for RL is the assumption that the samples are independently and identically distributed. Obviously, when the samples are generated from sequential exploration in an environment for the robot locomotion, this assumption is violated. To solve this, the replay buffer is used in DDPG. The replay buffer is a finite-size cache filled with transition samples. At each time step, both the actor network and the critic network are updated by sampling a mini batch uniformly from the buffer. Since DDPG is an off-policy learning algorithm, the replay buffer can be large where the algorithm benefits from a set of uncorrelated transitions. At each time step, the critic network θ^*Q*^ is updated by minimizing the loss:*

(18) $$L=\frac{1}{H}\sum_{h=1}(Y_{h}-Q(s_{h},a_{h}|\theta^{Q}){)}^{2},$$

where

(19) $$\begin{array}{r} Y_{h}=r_{h}+\gamma Q^{\prime }\left(s_{h+1},\pi^{\prime }\left(s_{h+1}|\theta^{\pi^{\prime }}\right)|\theta^{Q^{\prime }}\right), \end{array}$$

*and *h* is the time step. *H* is the size of the mini batch sample*.

The actor network θ^π^ is updated using the sampled policy gradient:

(20) $$\begin{array}{r} \nabla_{\theta^{\pi }}J=\frac{1}{H}\underset{h=1}{\sum }\nabla_{a}Q\left(s,a|\theta^{Q}\right){|}_{s=s_{h},a=\pi \left(s_{h}\right)}\nabla_{\theta^{\pi }}\pi \left(s|\theta^{\pi }\right){|}_{s_{h}}, \end{array}$$

### 4.3. Observation Space

The hexapod robot interacts with the environment through observations and actions. In order to apply DDPG on a practical system, the observation space is required to match the real robot and provides enough information for the agent to learn the task. In this work, a MDP observation vector *o*_*t*_ at time *t* is defined as:

(21) $$\begin{array}{r} o_{t}=<p_{b},v_{b},O,\theta ,\dot{\theta },\tau ,A>, \end{array}$$

where O is the body orientation. A is the policy output including the amplitude and phase difference in the limb layer of the CPG.

In addition, the observation space consists of only part of the states, so the MDP can not be fully described. For example, the hexapod robot can not identify terrain types without any use of exteroceptive sensors. Hence, the process is referred as a Partially Observable Markov Decision Process (POMDP). Since in our work, the hexapod robot interacts with the environment through a continuous trajectory rather than a discrete action, we find that our observation space is sufficient enough for learning the desirable tasks.

### 4.4. Action Space

The control policy outputs the coupling parameters of the limb layer of the CPG which determine the intra-limb coordination as well as the adaptation to different terrain types. The action space is defined as follows:

(22) $$\begin{array}{r} a_{t}=<\mu ,\theta_{i1}^{i2}>, \end{array}$$

The action vector is transmitted as the input of the CPG network which generates the joint positions for each joint actuators.

The two-layer CPG network is chosen as the locomotion generator instead of learning joint position commands directly like most of the other studies (Hwangbo et al., 2019; Tsounis et al., 2020). There are three reasons for this: (1) the CPG network constrains the basic locomotion of the robot, which reduces the search space and accelerates the learning; (2) compared to 18 joint position or joint torque commands, learning symmetric CPG coupling parameters lowers the dimension of the action space; (3) the CPG network outputs smooth joint position commands, which are easier to be realized in the real robot.

### 4.5. Network Architecture

**Figure 7:**
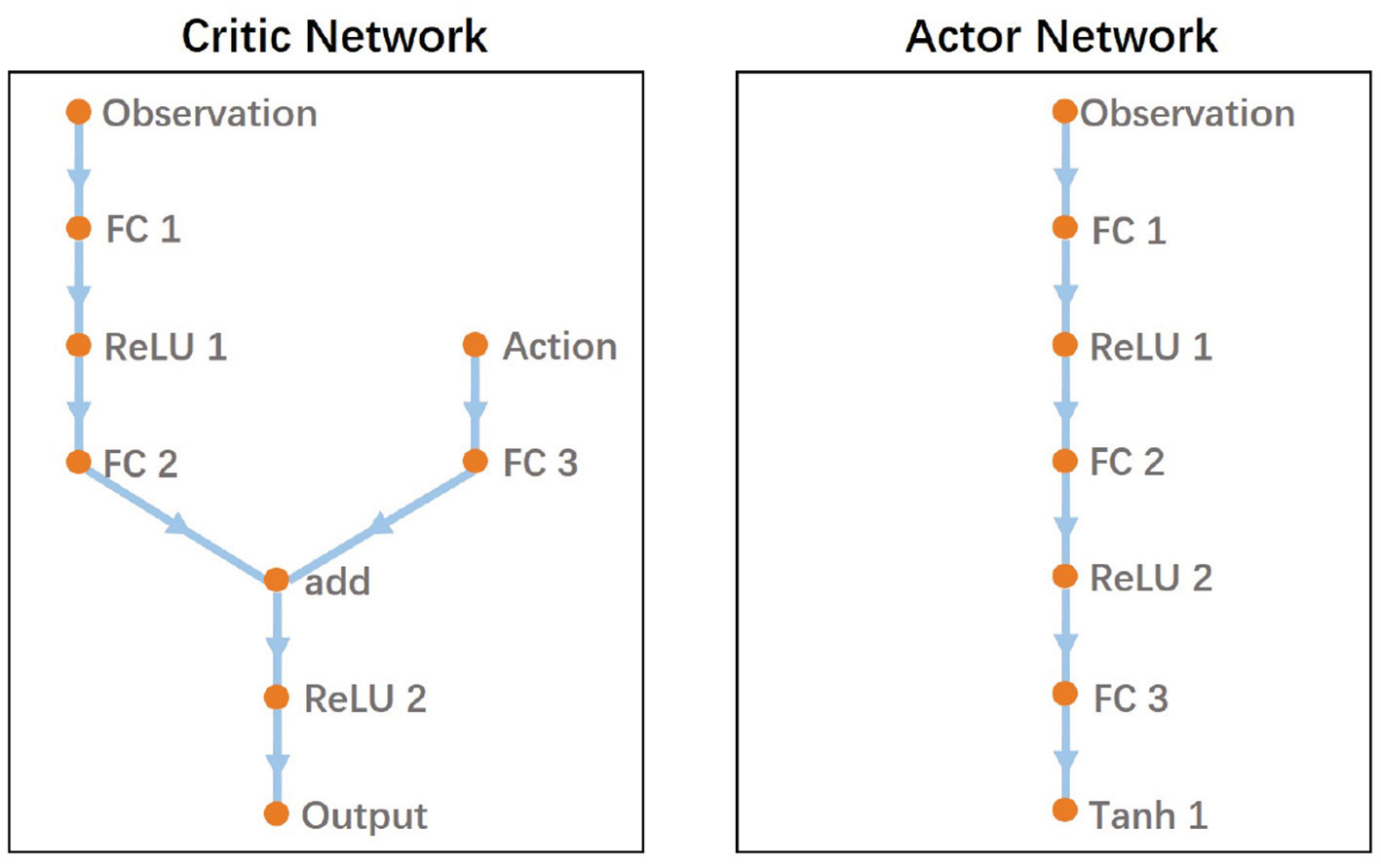
The network architecture of the proposed DDPG-based RL.

In DDPG, the critic network and actor network are parameterized as deep neural networks. Figure 7 provides a graphical depiction of the NN model. The model is similar to the network architecture implemented in Fujimoto et al. (2018) and is proved to perform well. The critic network is composed of five hidden layers including three fully-connected (FC) layers and two ReLU layers. The actor network consists of six hidden layers including three fully-connected (FC) layers, two ReLU layers and a Tanh layer. The output modulates the proposed two-layer CPG parameters.

### 4.6. Reward Function

In this work, the environmental adaptability of the hexapod robot is measured by two criteria: one is the heading velocity of the robot and the other is the energy consumption of the robot. In general, precise reward function in robotics is one of the main challenges in solving the RL problem. Due to the constraints in RL, the reward function is simplified to encourage faster heading velocity and penalize higher energy consumption.

Table 5 shows the detailed reward terms. The velocity reward term motivates the robot to move forward as fast as possible, and it is tuned so that the robot receives a reward for a positive velocity up to a certain point. The penalty term is used to optimize the energy consumption of the robot. Hence, the reward term and the penalty term is intergraded into the reward function *r*_*t*_ as follows:

(23) $$r_{t}=K_{v}\cdot v_{x}-K_{e}\cdot (|\tau \cdot \dot{\theta }|+\tau^{2}),$$

where *K*_*v*_ and *K*_*e*_ are the positive weights.

**Table 5:** Reward terms.

**Term**	**Expression**
Forward velocity	*v*_*x*_
Energy consumption	$|\tau \cdot \dot{\theta }|+\tau^{2}$

### 4.7. Guided Constrained Costs

In this work, two types of constrains are introduced into the proposed RL method (Gangapurwala et al., 2020). The first is the performance constraint, which restricts the hexapod robot into the region with potential good performance. The second is the safety constraint to avoid the robot exploring the region where damages may occur.

1. Performance Constraint Costs: These costs are directly added to the reward function, as shown in Table 6. The constraint costs are guided by the kinematic model of the hexapod robot and help to improve the locomotion performance. For example, the Joint Speed term and Torque term are the limits of the actuator performance of the robot. In our control scheme, each supporting leg of the hexapod robot moves symmetrically, so the Orientation term and Height term are given to limit the robot from swinging too much.
2. Safety Constraint Costs: For the cases when control policy outputs actions that cause the robot to land on unstable and unrecoverable states and damage the robot, the safety constraints are introduced in Table 7. The Fall term and Roll term are given to judge whether the robot falls or rolls over. If the control policy outputs the commands that robot can not carry on (see Joint Speed and Torque) or the robot falls and rolls over (see Fall and Roll), the training episode is terminated directly. The training steps explored in this episode are abandoned and a negative terminal reward is added to the last training step in the reformatted episode samples. This control policy avoids inefficient explorations of some constrained regions because the training episode is terminated if any safety constraint costs is true.

**Table 6:** Performance constraint costs.

**Term**	**Expression**
Joint speed	$||max\left(|\dot{\theta }|-{\dot{\theta }}^{max},0\right)|{|}^{2}$
Torque	||*max*(|**τ**|−**τ**^*max*^, 0)||^2^
Orientation	||O||^2^
Height	$||z_{c}-z_{c0}|{|}^{2}$

*${\dot{\theta }}^{max}$ and τ^max^ refer to the maximum outputs of the robot actuator. z_c_ and z_c0_ are the current and original body height of the hexapod robot. Orientation and Height limit the swing range of robot body*.

**Table 7:** Safety constraint costs.

**Term**	**Expression**
Joint speed	bool($\dot{\theta }>{\dot{\theta }}^{max}$)
Torque	bool(**τ** > **τ**^*max*^)
Fall	bool(*z*_*c*_ < 0)
Roll	bool(O > O^*limit*^)

*bool(·) is a general Boolean judgement function*.

## 5. Simulations and Experiments

The proposed bio-inspired learning scheme is used to shape the hexapod robot locomotion. We evaluate the feasibility and performance of the motion policy via four different terrains in both simulations and experiments.

### 5.1. Simulations

The aim of these simulations is to guarantee the convergence of RL algorithm and obtain the theoretical maximum velocity of the hexapod robot in forward motion under different terrains.

**Figure 8:**
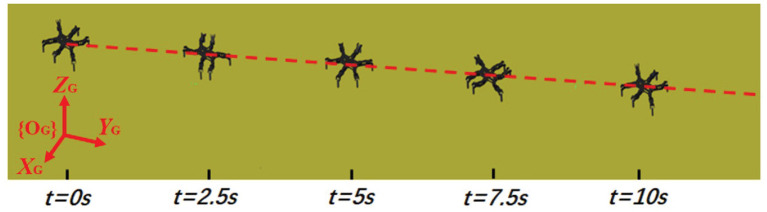
The RL training simulation in an episode.

**Figure 9:**
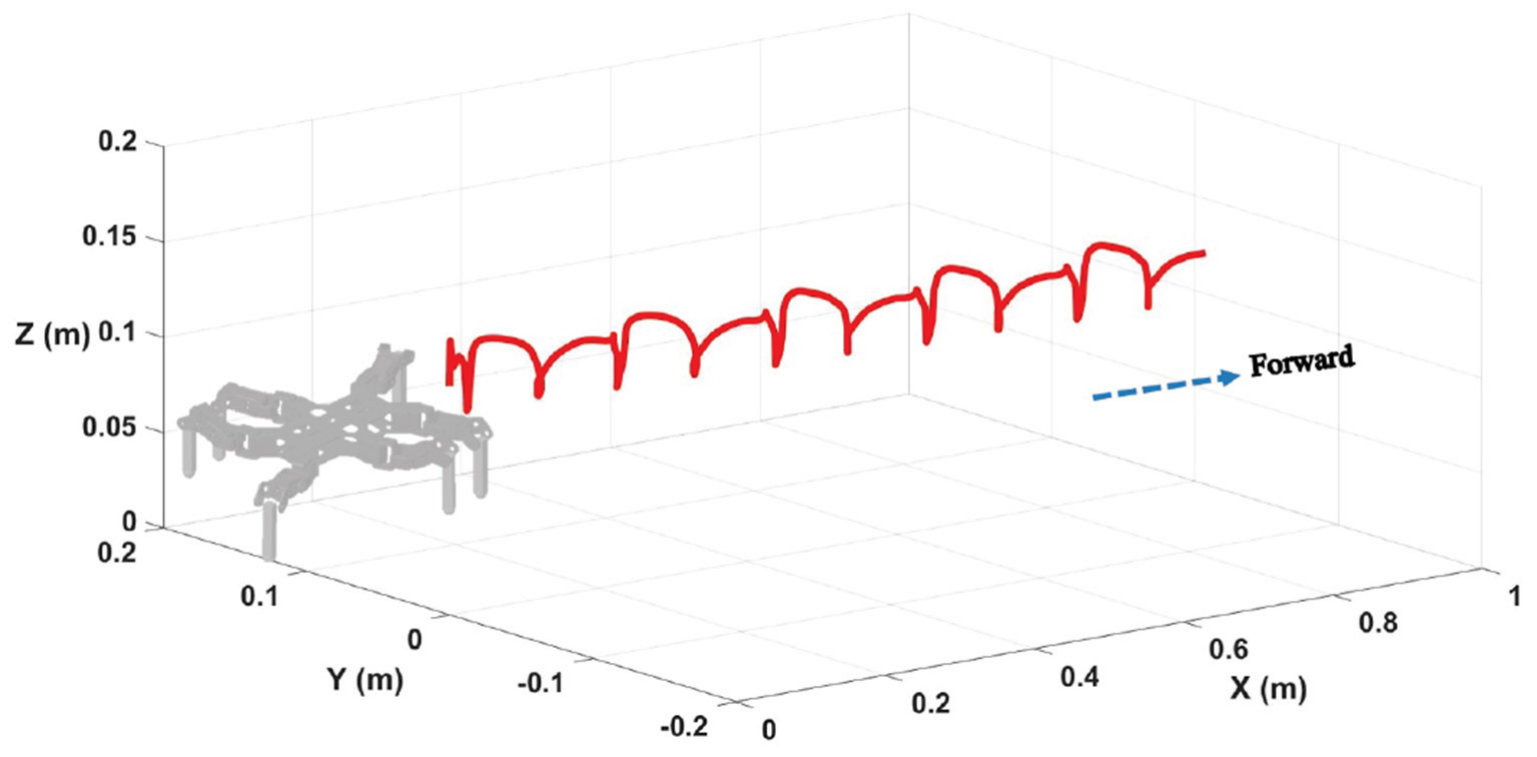
The motion trajectory of the hexapod robot.

The hexapod robot is modeled corresponding to the dimensions and mass of the actual hexapod robot prototype where the contact friction dynamics are determined by material surfaces. There are five main parameters for simulations: (1) learning rates for actor-critic network are set as 0.005 and 0.001, respectively; (2) the maximum number of episodes and steps in an episode are set as 1,400 and 200, respectively; (3) the sampling time is similar to the CPG cycle time which is 1 s; (4) the frequency of the proposed two-layer CPG network is fixed on 0.5. The contact friction coefficients are modified according to different ground materials. The training process of an episode is randomly chosen in Figure 8 and the motion trajectory is displayed in Figure 9.

From the two figures, it is noted that the hexapod robot walks well in the tripod locomotion without obvious offset in the direction of Y and Z axis. For a forward motion, we would like to emphasize that the sideslip in the vertical direction will cause an extremely uncertain deviation for the whole movement direction and posture. Therefore, the nearly tiny offset in Y axis illustrates the effectiveness of the proposed motion control scheme. As can be seen on Z axis, the slight fluctuation with the body height also reflects the control stability of the robot under the benefit of physical constraints.

**Figure 10:**
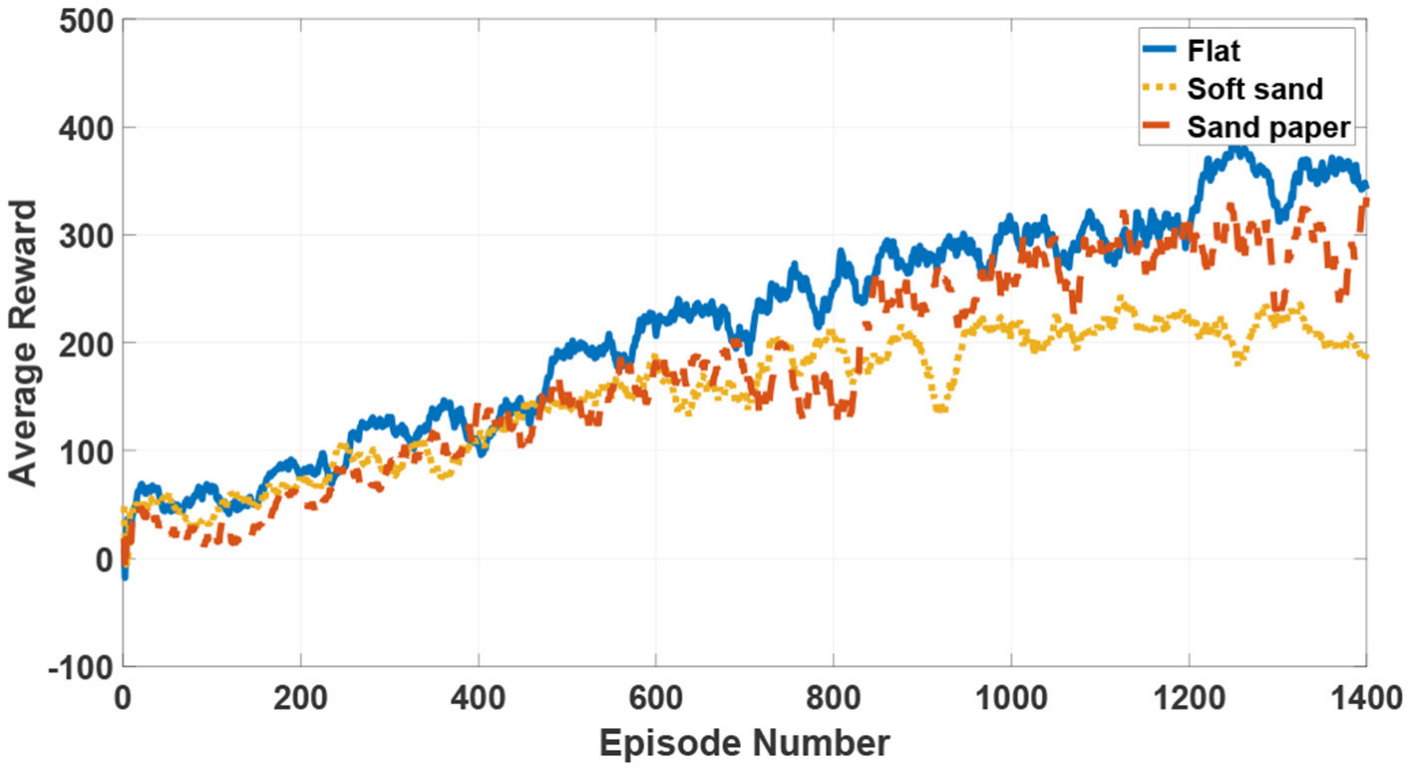
The average reward of reinforcement learning in the tripod locomotion.

In the training process, the observations are acquired from the hexapod dynamic model and the actions directly modulate the amplitude μ and phase difference $\theta_{mn}^{ij}$ in the limb layer of the CPG network. The reward function is given in the aforementioned section. At the end of 1,400 episodes, the average reward converges to a stable value in three terrain types as shown in Figure 10. The average training time in these terrains is approximately 6 h.

During learning processes, zero initial values drive the hexapod robot to swing around the origin and the value of reward function is equal to zero. After postures adapting and actions updating, the robot locomotion continuously becomes smoother. As the motion stability performance is improved, the reward value increases over time. Finally, the accumulative data samples help the robot reach the best motion state under the specific locomotion mode and the reward function also converges to the biggest value. Compared with the movements in a sand paper and a soft sand, the steady velocity in a flat environment is a bit faster, but the convergence rate is conversely slower. As can be seen from the whole learning episodes, there are no obvious differences of the learning trend among three terrains. Besides, although the learning processes may suffer from several asymmetric and non-natural looking, even defective locomotion, the hexapod robot will finally converge to a stable and optimal locomotion under the limitation of several presetting constraints in the proposed DDPG-based learning approach.

**Figure 11:**
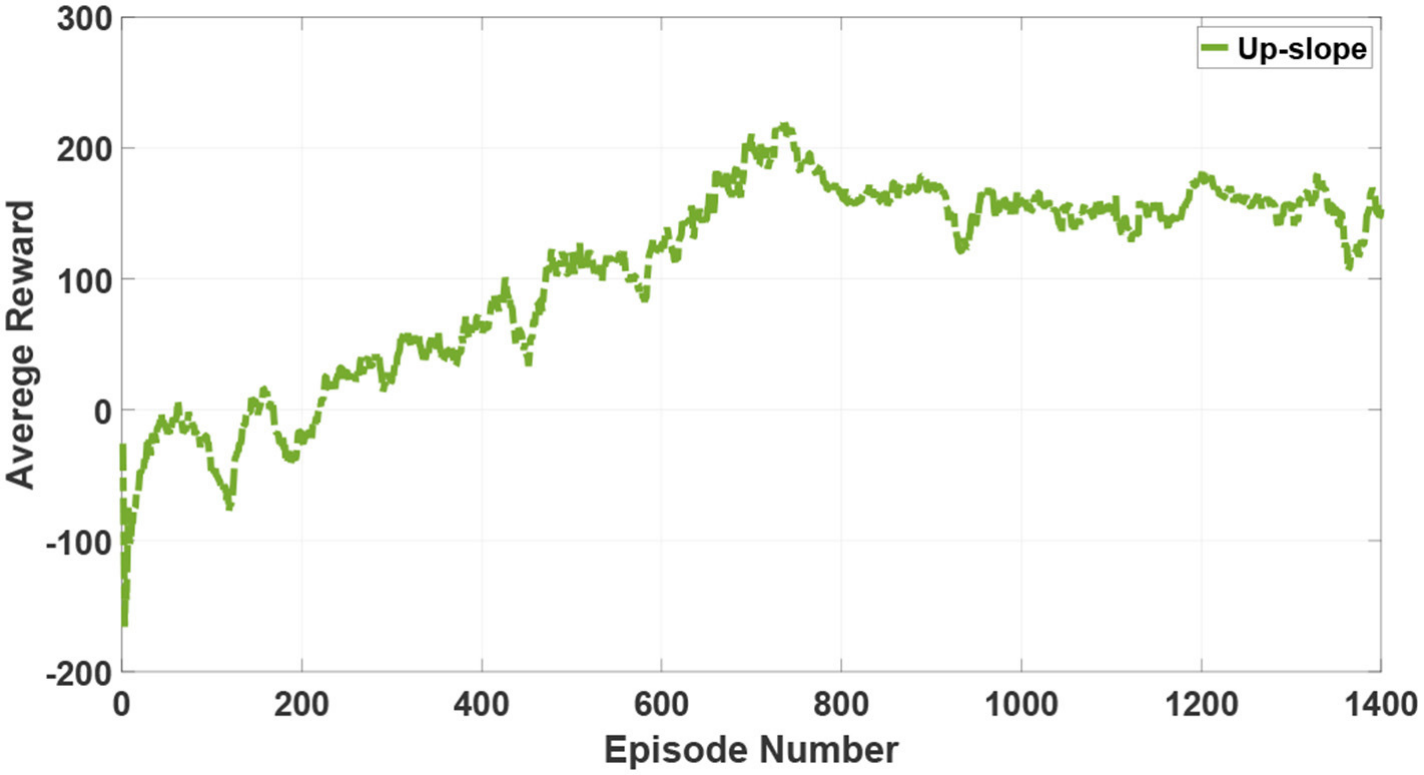
The average reward of reinforcement learning in the quadruped locomotion.

As mentioned in section 3, tripod locomotion can be the fastest but inflexible. Therefore, when encountering complex and harsh terrains such as a slope or stones, the robot will switch to flexible locomotion modes such as quadruped locomotion or five legs support locomotion. In order to exhibit the locomotion flexibility derived from the proposed 3D two-layer CPG controller, an up-slope (10 deg) terrain is simulated and the quadruped locomotion is generated for repeating the aforementioned training process. The result of the average reward is represented in Figure 11.

Although the hexapod robot also accomplishes a fast convergence in an up-slope after 1,400 episodes, the average reward in such a tougher terrain is obviously lower than the stable value in a flat. Moreover, based on excellent adjustment characteristics of the proposed 3D two-layer CPG controller, the hexapod robot is endowed with the capability of locomotion transitions for adapting to complicated and unstructured terrains.

### 5.2. Experiments

Similarly, four experiments on different terrain surfaces, namely, flat, sand paper, soft sand, and up-slope, are conducted to validate the adaptivity and robustness of the proposed bio-inspired learning approach in practical scenarios. The training time is set as 5 s in each episode and other parameters set in these experiments are the same as simulation parameters. In addition, the simulation results can reduce the experimental training time through offering the hexapod robot an initial policy that performs the best in the simulations.

**Figure 12:**
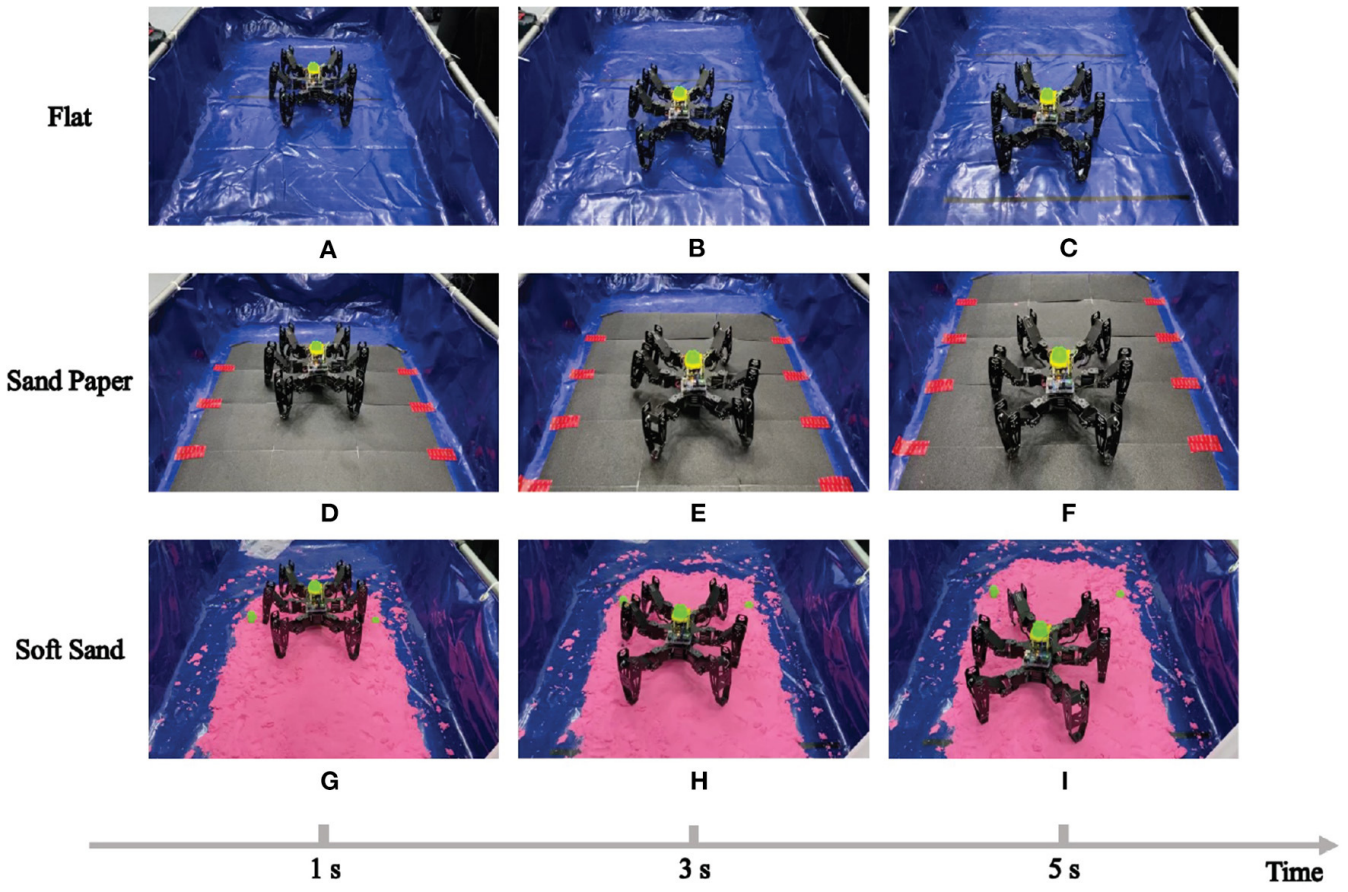
The RL training experiment in different terrains.

Firstly, environmental adaptability under individual locomotion mode has been tested. Here, the most common locomotion, tripod locomotion, is adopted as the training mode in three different terrain surfaces. The neural network in RL-based algorithm evolves three corresponding policies to make the robot perform well on the specific surfaces. The experiment scenes are shown in Figures 12A–I, where the same robot crawls on different contact surfaces.

During the repetitive episodes in the three mentioned terrains, the actual velocity of the hexapod robot with regard to the CPG tuning parameters, namely, the amplitude μ and phase difference $\theta_{mn}^{ij}$ in the limb layer are recorded. All these raw data are fitted and the learning results are shown in **Figures 14A–C**.

As can be seen from these figures, the results in different terrains have similar characteristic (the convex surface), but there are obvious differences in specific nodes. For example, when the amplitude is 1 with phase difference is 0.1, the velocity of the robot crawling on the flat will drop significantly from its maximum speed, but it decreased slowly on the soft sand.

**Figure 13:**
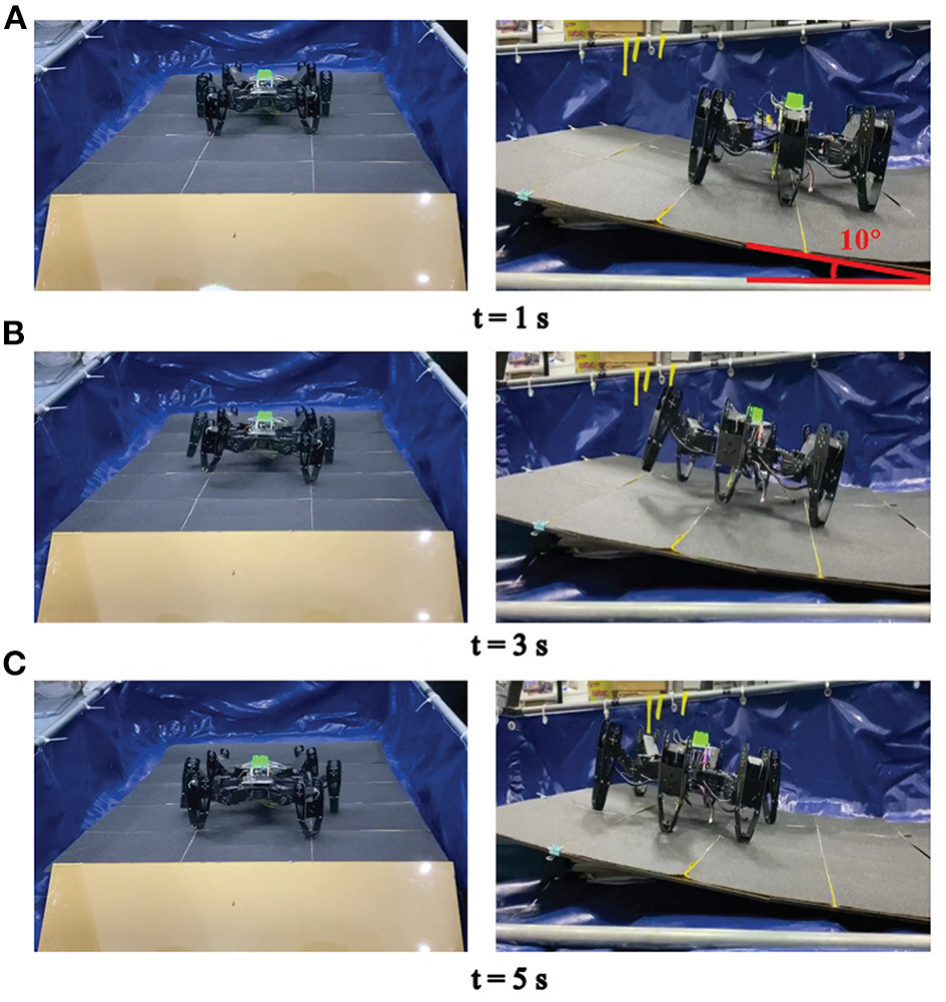
The RL training experiment in an up-slope.

**Figure 14:**
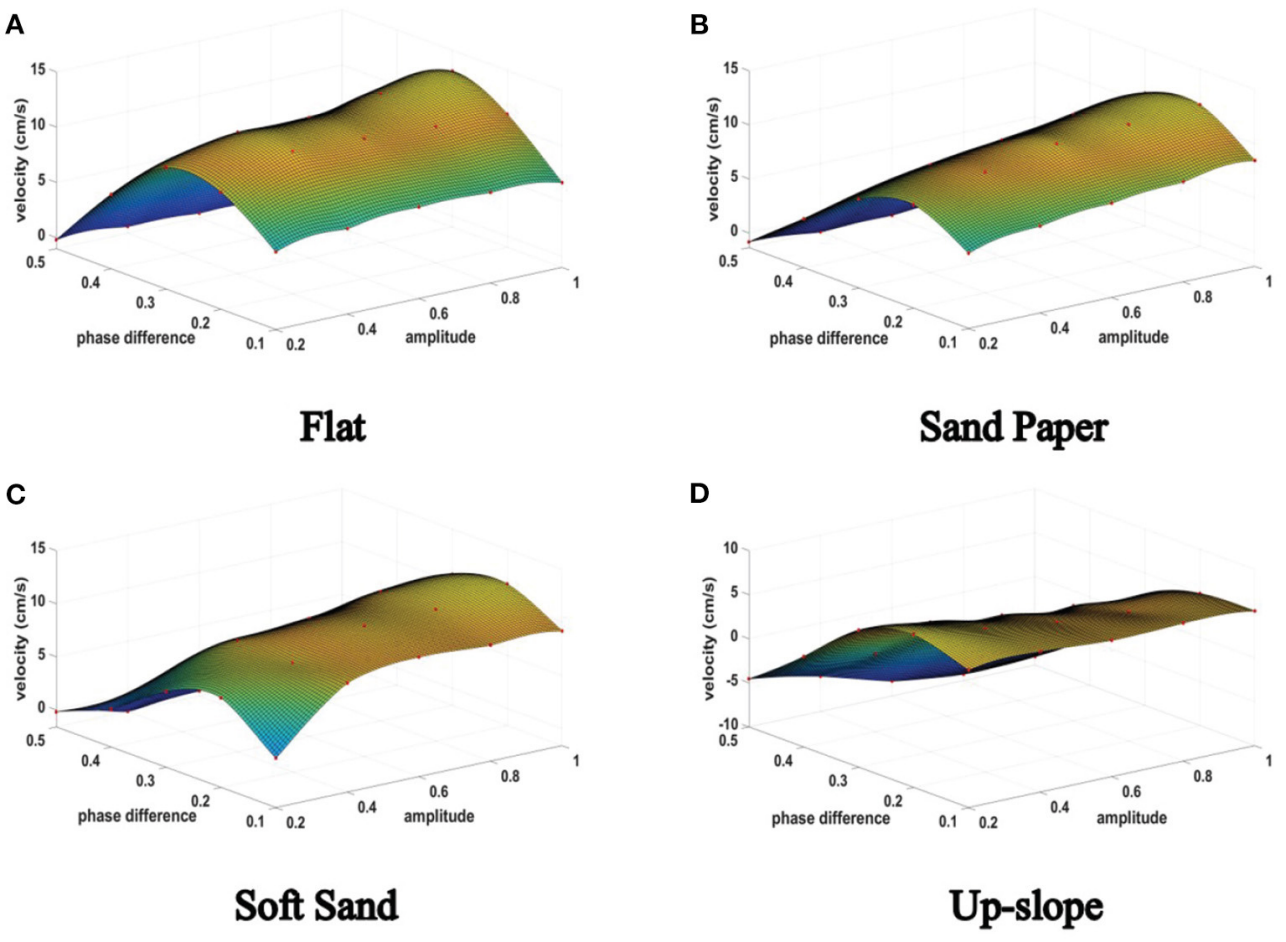
The hexapod robot velocity in different terrains.

Next, the quadruped locomotion in an up-slope (10 deg) is trained to evaluate the adaptability of different locomotion mode generated by the 3D two-layer CPG network (especially the body layer). The experimental platform and the learning result are illustrated in Figures 13A–C, 14D, respectively. As observed in this experiment, the quadruped locomotion performs well and stably in up-slope environment showing a different trend compared with tripod locomotion, which explains the impact of basic locomotion patterns to the robot behavior.

Finally, the maximum velocities in the four terrains are calculated as listed in Table 8. It is noticed from Table 8 that the hexapod robot runs fastest on the flat under tripod locomotion while it runs slowest under quadruped locomotion on the up-slope. Compared with the body length (BL) of the hexapod prototype (24 cm), the maximum velocity is 1/3∽1/2 BL from 7.35 to 13.10 (cm/s) in the preset terrains. As can be seen, since the surface friction coefficients of the chosen Sand Paper and Soft Sand terrain belong to the category of sand which may be similar to some extent, the difference of maximum velocity is not obvious.

**Table 8:** The maximum velocity in different terrains.

**Terrain types**	**Velocity(cm/s)**	
	**Simulations**	**Experiments**
Flat	22.27	13.10
Sand paper	19.87	11.86
Soft sand	15.40	11.72
Up-slope	13.05	7.35

It should be also emphasized that, suffering from inaccuracies in modeling process as well as environment construction uncertainties, several deviations between simulation model and actual experiment exist inevitably. For instance, the parametric variables in simulation are fixed, while all the variables are inherently floating in the experiments. Nevertheless, the simulation results still have a certain association with the experimental results, which effectively offer prior information at the beginning of experiment settings and greatly accelerate the convergence rate in the actual system.

In summary, it can be concluded from the experimental results that the proposed 3D two-layer CPG network and the DDPG-based RL algorithm can provided the hexapod robot with excellent maneuverability and environmental adaptability performance while the stability and robustness of the overall control scheme can be also achieved.

## 6. Conclusion

This paper aims to investigate an adaptive locomotion control approach for a hexapod robot. Inspired by biological neuron control systems, the proposed locomotion controller is composed of a set of coupled oscillators, namely an artificial CPG network. The novelty of the CPG controller lies in its 3D two-layer. The first layer of the CPG is able to control the basic locomotion patterns according to the environment information, while a RL-based learning algorithm is adopted for fine-tuning the second layer of the CPG to regulate the behavior of robot limbs. Several numerical studies and experiments have been conducted to demonstrate the valid and effectiveness of the proposed locomotion controller. The navigation of the robot in a complex and dynamic environment will be explored in the next research phase.

## Data Availability Statement

The original contributions presented in the study are included in the article/supplementary material, further inquiries can be directed to the corresponding author/s.

## Author Contributions

All authors contributed to the theory and implementation of the study. WO designed the whole locomotion control scheme, proposed the two-layer CPG, and wrote the first draft of the manuscript. HC modeled the hexapod robot and carried on the experiments. JP offered the simulation of the reinforcement learning part. WL corrected the paper formation and text required for the journal. QR determines the final Abstract, Introduction, and Conclusion. All authors contributed to manuscript revision, read, and approved the submitted version.

## Conflict of Interest

The authors declare that the research was conducted in the absence of any commercial or financial relationships that could be construed as a potential conflict of interest.
